# Supplementary material for: Choroid plexus NKCC1 mediates cerebrospinal fluid clearance during mouse early postnatal development
Source: Nat Commun. 2021 Jan 19;12:447. doi: 10.1038/s41467-020-20666-3 (PMC7815709; doi:10.1038/s41467-020-20666-3)
Supplement: Supplementary file 1 — Supplementary Information [file 41467_2020_20666_MOESM1_ESM.pdf]

Choroid plexus NKCC1 mediates cerebrospinal fluid clearance during mouse early postnatal development

Huixin Xu[1,‡], Ryann M Fame[1,‡], Cameron Sadegh[1,2], Jason Sutin[3], Christopher Naranjo[4], Della Syau[4], Jin Cui[1], Frederick B Shipley[1,5], Amanda Vernon[6], Fan Gao[6,†], Yong Zhang[7], Michael J. Holtzman[7], Myriam Heiman[6], Benjamin C Warf[8], Pei-Yi Lin[3], Maria K Lehtinen[1,5 *]

SUPPLEMENTAL INFORMATION

[1]Department of Pathology, Boston Children's Hospital, Boston, MA 02115, USA

[2]Department of Neurosurgery, Massachusetts General Hospital and Harvard Medical School, Boston, MA 02114, USA

[3]Fetal-Neonatal Neuroimaging and Developmental Science Center, Division of Newborn Medicine, Boston Children's Hospital, Harvard Medical School, 300 Longwood Avenue, Boston, MA 02115, USA

[4]Summer Honors Undergraduate Research Program, Division of Medical Sciences, Harvard Medical School, Boston, MA 02115, USA

[5]Graduate Program in Biophysics, Harvard University, Cambridge, MA 02138, USA

[6]Broad Institute of MIT and Harvard, Cambridge, MA 02142; Picower Institute for Learning and Memory, Cambridge, MA 02139; Department of Brain and Cognitive Sciences, Massachusetts Institute of Technology, Cambridge, MA 02139, USA

[7]Pulmonary and Critical Care Medicine, Department of Medicine, Washington University, St. Louis, MO 63110, USA

[8]Department of Neurosurgery, Boston Children's Hospital, Boston, MA 02115, USA

[‡]These authors contributed equally.

[†]Current address: Bioinformatics Resource Center in the Beckman Institute at Caltech, Pasadena, CA 91125, USA

*Correspondence should be addressed to: maria.lehtinen@childrens.harvard.edu

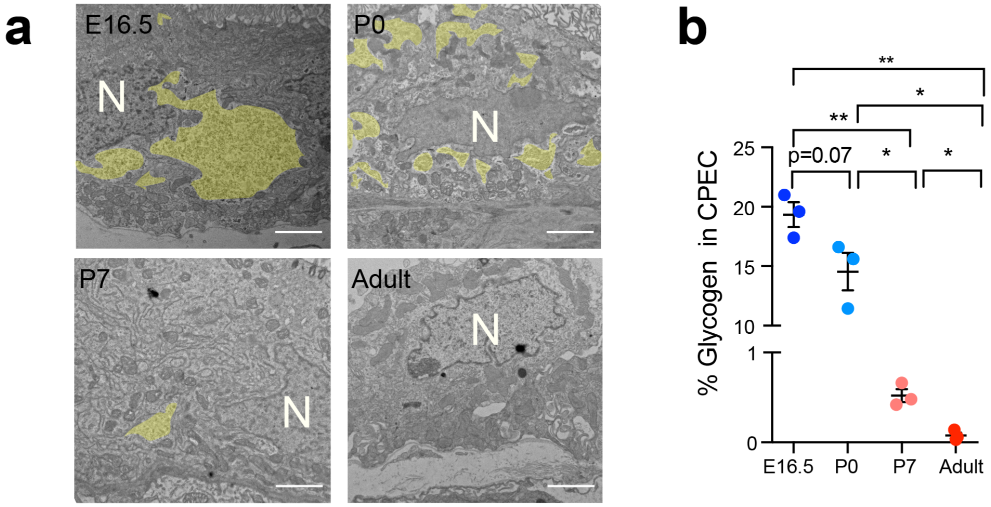

Supplementary Fig. 1. Glycogen load in ChP epithelial cells. a Representative transmission electron micrographs of E16.5, P0, P7, and adult LV ChP. Glycogen granules are highlighted in yellow. Scale bar = 2 µm. N = Nucleus. **b** Proportion of TEM fields of view that are filled with glycogen granules, N=3 animals from 2 independent experiments (same animals as those used in Fig. 1d-f), 10-15 fields of view (FOV) per animal (See source data for exact numbers of FOV), distinct cells were captured in each FOV., Blue to red color change represents age change, with blue being the youngest (embryonic) and red the oldest (adult). E16 vs. P7: ** p = 0.0030; E16 vs. adult: ** p = 0.0029; P0 vs. P7:* p = 0.0124; P0 vs. adult * p = 0.0117; P7 vs. adult * p = 0.0136; Welch's two-tailed unpaired t-test. CEPC, choroid plexus epithelial cell. Source data are provided as a Source Data file.

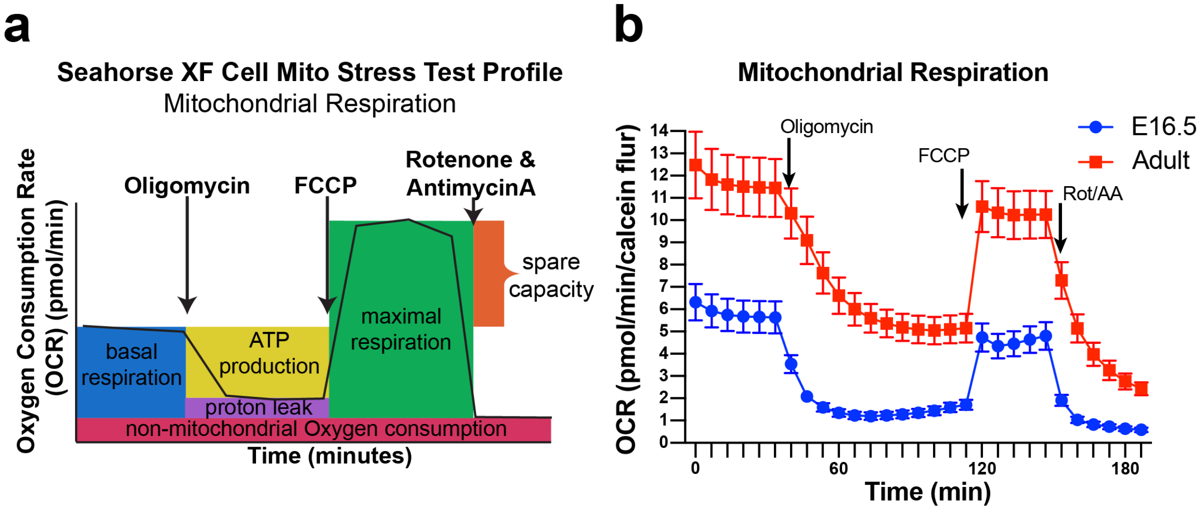

Supplementary Fig. 2. Seahorse XF Cell mito stress test profile and representative curves. a Schematic of the Agilent Cell Mito Stress Test showing the experimental design to quantify mitochondria basal respiration and ATP production. **b** Representative experiment of ChP in Cell Mito Stress Test; N = 12 E16.5 animals and N = 4 adult animals. Red: adult; blue: E16.5. Source data are provided as a Source Data file.

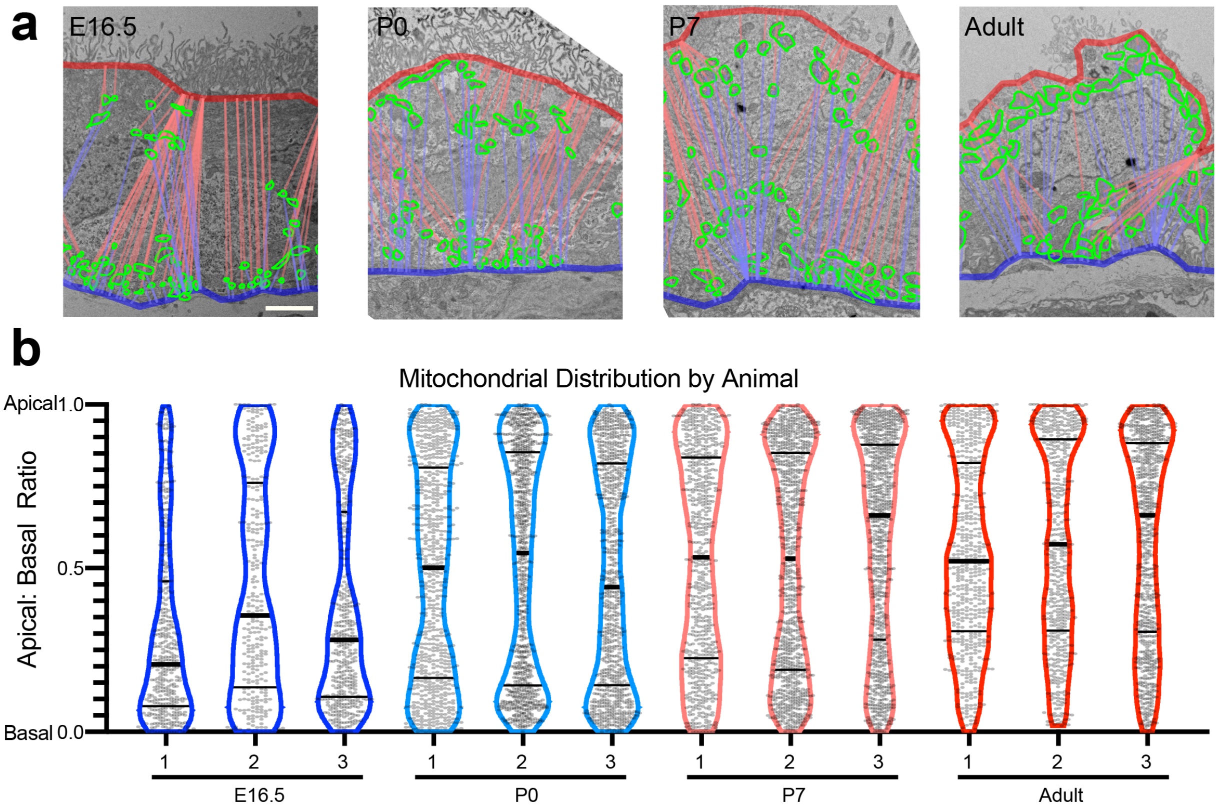

Supplementary Fig. 3. Representative images and quantification of ChP epithelial mitochondria distribution analysis. a Representative transmission electron micrographs of E16.5, P0, P7, and adult LV ChP. Mitochondria (green circle), apical membrane (red line), and basal membrane (blue line) are labeled. Scale bar = 2 μm. Images are representative of 2 independent experiments with a total of 3 biologically independent animals at each age. **b** Mitochondrial distribution plots from each animal (same as described in panel a). Apical: basal ratio: 1 is touching the apical surface and 0 is touching the basal surface. Solid thick line indicates median and thinner line indicates upper/lower quartiles. Blue to red color change represents age change, with blue being the youngest (embryonic) and red the oldest (adult). Source data are provided as a Source Data file.

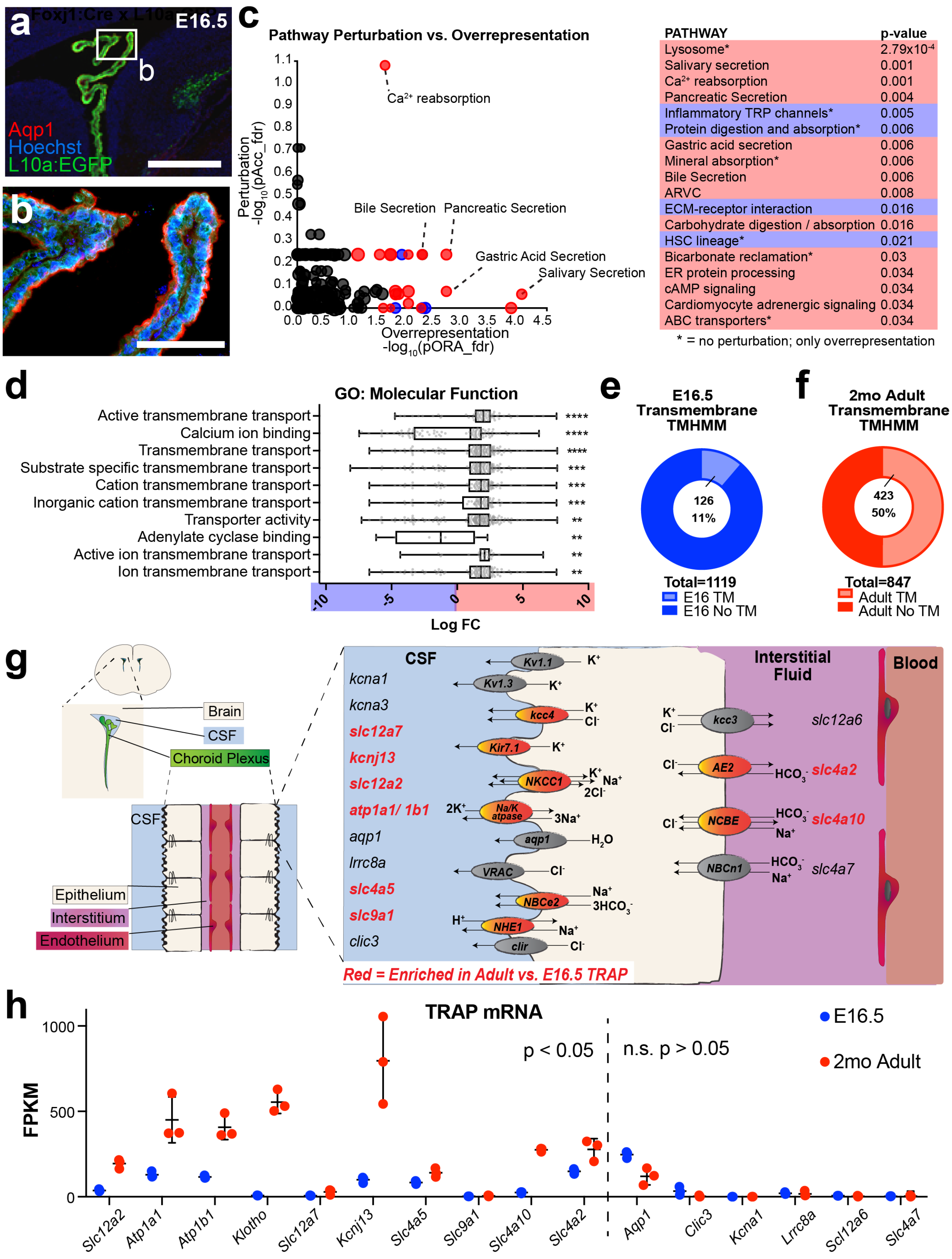

PATHWAY	p-value
Lysosome*	2.79×10^{-4}
Salivary secretion	0.001
Ca^{2+} reabsorption	0.001
Pancreatic Secretion	0.004
Inflammatory TRP channels*	0.005
Protein digestion and absorption*	0.006
Gastric acid secretion	0.006
Mineral absorption*	0.006
Bile Secretion	0.006
ARVC	0.008
ECM-receptor interaction	0.016
Carbohydrate digestion / absorption	0.016
HSC lineage*	0.021
Bicarbonate reclamation*	0.03
ER protein processing	0.034
cAMP signaling	0.034
Cardiomyocyte adrenergic signaling	0.034
ABC transporters*	0.034

Supplementary Fig. 4. Supportive analysis of TRAP sequencing. a-b Rpl10a-conjugated EGFP expression in ChP epithelial cells after *Foxj1*-Cre recombination in TRAP-BAC mice. Aqp1 marked ChP epithelial apical membrane. Scale bars = 500 μm (a) and 100 μm (b). Representative of 2 experiments, each with 2 biologically independent replicates. **c** Perturbation vs. overrepresentation analysis via iPathway (Advaita) reveals enriched pathways at E16.5 (blue) and Adult (red) (the same red vs. blue color scheme is used for the rest of this figure). * indicates pathways that are only overrepresented, but not predicted to be additionally perturbed at the network level. **d** Top 10 significantly enriched GO terms for "molecular function". Plotted with center bars as median, bounds of boxes for quartiles, and whiskers for maximum and minimum values. The $\log_{10}$ fold change (LogFC) is plotted for each expressed gene for the network. Positive values (red) indicate Adult enrichment and negative values (blue) indicate E16.5 enrichment. *p* values are corrected for multiple measures using Bonferroni correction. See Supplementary Data S1 for exact *p* - values; $^{**}p \leq 0.01$, $^{***}p \leq 0.001$, $^{****}p \leq 0.0001$. **e-f** Proportion of enriched genes in E16.5 (blue) and Adult (red) ChP with predicted transmembrane domains using TMHMM. **g** Schematic of ChP localization within brain ventricles, relative position to blood and CSF, and transporters. Red highlights: significantly enriched in Adult vs. E16.5 TRAP (adjusted $p < 0.05$). **h** FPKM values from TRAP of transcripts associated with ChP transport.

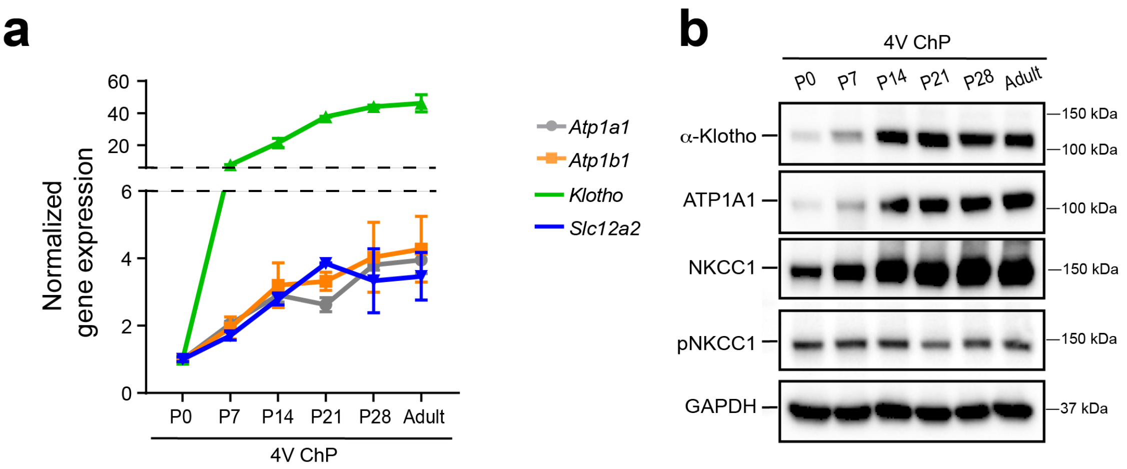

Supplementary Fig. 5. TRAP candidate validation in 4V ChP. a-b RT-qPCR (N=4 biologically independent animals from two experiments for each timepoint, colors were chosen to match with Fig. 2f) and immunoblotting of 4V ChP during postnatal development, representative of 3 independent experiments, each with tissues from 1-2 animals pooled (2 animals for ages under P14, 1 animal for ages of P14 and older) for each timepoint Source data are provided as a Source Data file.

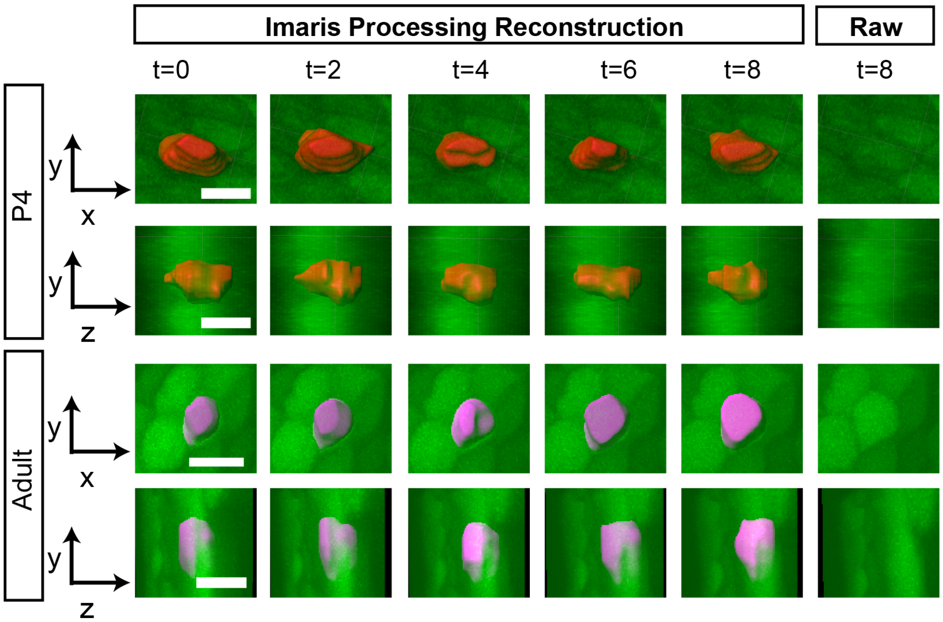

Supplementary Fig. 6. Workflow of IMARIS demonstrating the cell volume quantification process. At each time point, the reconstructed 3D cell mask is highlighted (P4 in red and adult in pink) and views from x-y plane and y-z plane are displayed. Raw images from a single plane at the last time point are shown on the right end. Scale bar = 10 μm (P4) and 50 μm (Adult). A total of 3 independent experiments were conducted, each contained 2-3 biological replicates, only tissues with good quality (i.e. tissues without tears or appearing stretched/crumpled due to mounting, and with good calcein signal indicating viability) were included in quantification, resulting in N=4 for each age.

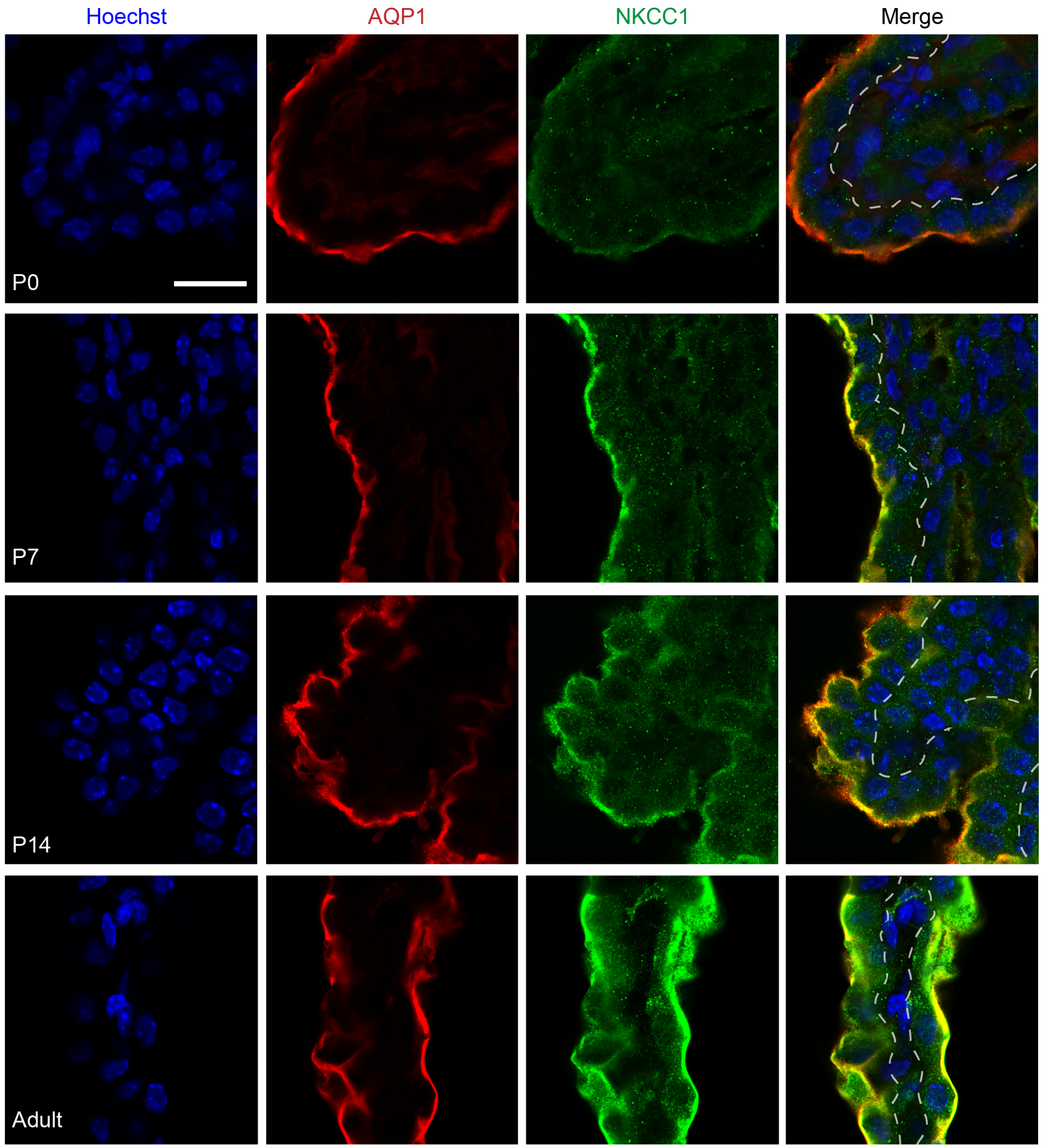

Supplementary Fig. 7. Localization of NKCC1 (green) at the apical membrane of ChP epithelial cells across development (P0, P7, P14, and Adult). AQP1 (red) marks the apical membrane; the white dashed line marks the basal membrane. Scale bar = 20 µm. N = 4 biological replicates were included for each age, from 2 independent experiments.

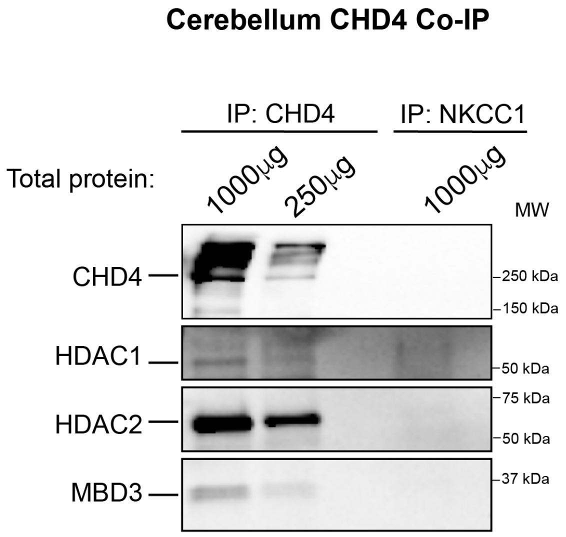

Supplementary Fig. 8. Co-IP immunoblots using adult mouse cerebellum to validate the Co-IP protocol, representative of 3 independent experiments, each contained tissues collected from 1-2 mice. An antibody targeting CHD4 co-immunoprecipitated several other complex members including HDAC1, HDAC2, and MBD3, from mouse cerebellar lysate, while a negative control performed with a control antibody of the same host species (in this case anti-NKCC1 antibody) failed to pull down NuRD-CHD4 complex members. Source data are provided as a Source Data file.

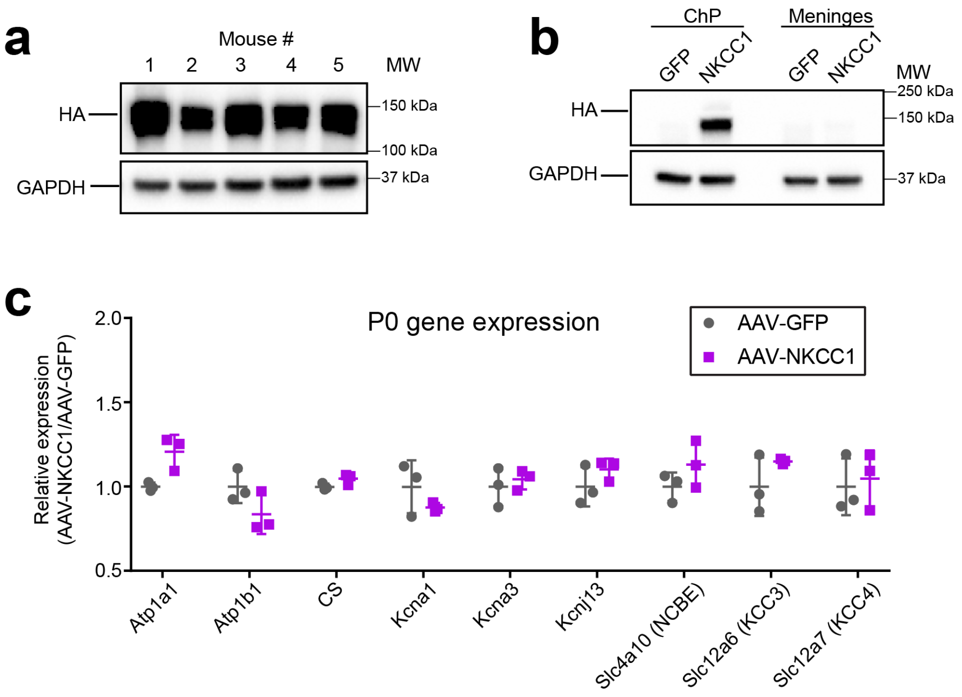

Supplementary Fig. 9. Validation of AAV2/5-NKCC1 transduction efficiency and specificity. a Immunoblots of AAV2/5-NKCC1 transduced ChP showing successful but variable transduction rate within one litter. N=5 biologically independent animals collected from two experiments. **b** Immunoblot of AAV transduced ChP and meninges showing non-detectable meningeal transduction by AAV2/5-NKCC1. Representative of 2 experiments, each with 2 biologically independent replicates. **c** RT-qPCR analysis of all other K^+ transporters and channels in the ChP after *in utero* viral transduction, showing no significant changes; Grey: AAV-GFP; purple: AAV-NKCC1. $\alpha > 0.05$, multiple t-Test corrected for multiple comparisons using the Holm-Sidak method. N=3 biologically independent animals collected from 2 experiments. Source data are provided as a Source Data file.

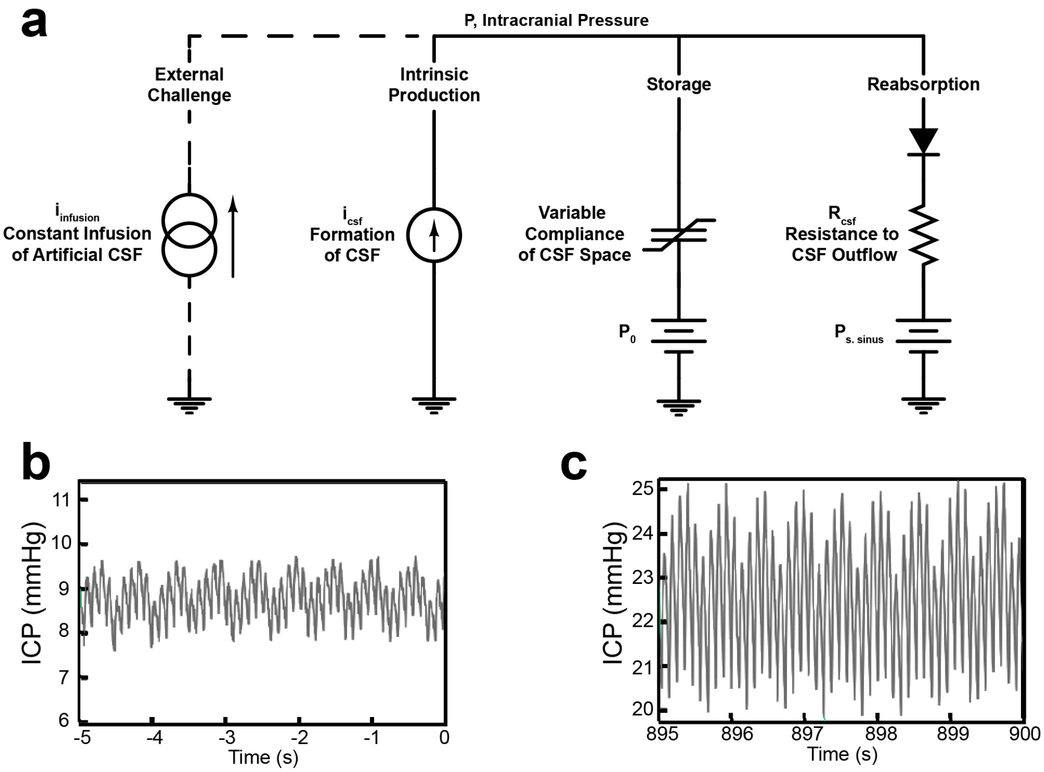

Supplementary Fig. 10. Mechanisms of constant CSF infusion test by Marmarou's model. a Marmarou's model of CSF dynamics. In this model, the physiologic processes of the cycle of CSF turnover are represented by analogous electric circuit elements, with ICP expressed as a solution to the circuit model in terms of lumped parameters describing the net effect of the processes on the level of ICP without attributing them to specific microscopic pathways. At the most basic level, the model is a statement of conservation of mass, with the rate of CSF production balanced by the rate of CSF storage in intracranial and spinal compartments plus the rate of CSF reabsorption. **b-c** Higher magnification of the ICP data of infusion test showing the normal arterial and respiratory components of the ICP waveform at the beginning of the test (**b**). The increase in waveform amplitude with ICP is expected with increasing volume load (**c**).

Supplementary Table 1. CSF ion concentrations at developmental stages. *p*: statistical comparison to adult values. * $p < 0.05$; ** $p < 0.01$; *** $p < 0.001$; **** $p < 0.0001$; ns = not significant.

	K^+				Na^+				Cl^-				Ca^{2+}				Mg^{2+}			
	**mM**	***p***		**N**	**mM**	***p***		**N**	**mM**	***p***		**N**	**mM**	***p***		**N**	**mM**	***p***		**N**
**E14.5**	7.969 ± 1.90	****	<0.0001	8	141.1 ± 6.10	ns	0.1851	5	104.9 ± 5.28	**	0.0054	5	3.42 ± 0.47	*	0.0205	3	1.76 ± 0.07	****	<0.0001	4
**P0**	9.590 ± 3.50	**	0.0015	5	126.2 ± 11.84	ns	0.03605	4	104.0 ± 3.27	**	0.0078	4	3.70 ± 0.84	*	0.0456	3	1.07 ± 0.04	***	0.0002	4
**P4**	4.903 ± 0.47	**	0.0013	4	131.9 ± 4.52	ns	0.1	4	111.0 ± 2.00	*	0.035	4	2.63 ± 0.21	ns	0.1273	3	1.09 ± 0.01	****	<0.0001	4
**P7**	4.363 ± 0.92	*	0.0348	4	144.5 ± 6.65	ns	0.8313	4	125.0 ± 2.00	ns	0.7499	4	3.13 ± 0.39	*	0.0328	3	2.04 ± 0.19	**	0.001	4
**P14**	3.283 ± 0.18	ns	0.6672	4	137.3 ± 3.22	ns	0.2686	4	115.1 ± 6.00	ns	0.1124	4	2.51 ± 0.36	ns	0.3443	4	0.53 ± 0.05	****	<0.0001	4
**Adult**	3.142 ± 0.61	/	/	6	146.2 ± 14.40	/	/	5	127.1 ± 12.01	/	/	5	2.24 ± 0.28	/	/	3	0.89 ± 0.04	/	/	5

Supplementary Table 2. Summary of publications reporting various values of ChP epithelium intracellular Na^+, K^+, Cl^- concentrations. (N.D., not determined).

**Publication**	**Species**	**Age**	**$[Na^+]_i$**	**$[K^+]_i$**	**$[Cl^-]_i$**
Gregoriades, J. M. C., Madaris, A., Alvarez, F. J. & Alvarez-Leefmans, F. J. Genetic and pharmacological inactivation of apical Na(+)-K(+)-2Cl(-) cotransporter 1 in choroid plexus epithelial cells reveals the physiological function of the cotransporter. *American journal of physiology. Cell physiology* 316, (2019).	Mouse	ChP epithelial culture collected from P10-21 mice	*9.2 ± 2.5mM*	*ND*	*60.7 ± 12.3mM*
Steffensen, A. B. *et al.* Cotransporter-mediated water transport underlying cerebrospinal fluid formation. *Nat Commun* 9, (2018).	Mouse	8-12 weeks	*31 ± 5mM*	*141 ± 12mM*	*35 ± 9mM*
-Keep, R. F., Xiang, J. & Betz, A. L. Potassium cotransport at the rat choroid plexus. *The American journal of physiology* 267, (1994).<br><br>-Zeuthen, T. The effects of chloride ions on electrodiffusion in the membrane of a leaky epithelium. Studies of intact tissue by microelectrodes. *Pflugers Archiv : European journal of physiology* 408(3), (1987).	Necturus maculosus	Mature	*30.0mM*	*119mM*	*50mM*
Johanson, C. E. & Murphy, V. A. Acetazolamide and insulin alter choroid plexus epithelial cell [Na+], pH, and volume. *The American journal of physiology* 258, (1990).	Rat	Adult	*48 ± 0.7mmol/kg*	*95 ± 1.2 mmol/kg*	*62 ± 0.3 mmol/kg*
Saito, Y. & Wright, E. M. Regulation of intracellular chloride in bullfrog choroid plexus. *Brain Res* 417, (1987).	Bullfrog	Mature	*10.5mM*	*ND*	*24mM*
