# Supplementary material for: Choroid plexus NKCC1 mediates cerebrospinal fluid clearance during mouse early postnatal development
Source: Nat Commun. 2021 Jan 19;12:447. doi: 10.1038/s41467-020-20666-3 (PMC7815709; doi:10.1038/s41467-020-20666-3)
Supplement: Supplementary file 3 — Description of Additional Supplementary Files [file 41467_2020_20666_MOESM3_ESM.pdf]

Description of Additional Supplementary Files

Title: Supplementary Data 1.

Description: Pathway analysis of TRAP Data. (Tab1) Adult Functional annotation clustering. (Tab2) E16 Functional annotation clustering. (Tab3) Adult Gene functional classification. (Tab 4) E16 Gene functional classification. (Tab 5) iPathway GOBiological Processes (Tab 6) I Pathways GO-molecular function (Tab 7) iPathways GO- Cellular Component (Tab 8) iPathways Pathway Enrichment.

Title: Supplementary Data 2.

Description: SignalP and TMHMM analysis of TRAP Data (Tab1) Adult SignalP output (Tab2) Adult SignalP gene list (Tab3) Adult SignalP DAVID Functional Annotation Clustering. (Tab 4) Adult TMHMM. (Tab 5) Adult SignalP without TM domain. (Tab6) E16 SignalP output (Tab7) E16 SignalP gene list (Tab8) E16 SignalP DAVID Functional Annotation Clustering. (Tab 9) E16 TMHMM. (Tab 10) E16 SignalP without TM domain
