# Supplementary material for: Choroid plexus NKCC1 mediates cerebrospinal fluid clearance during mouse early postnatal development
Source: Nat Commun. 2021 Jan 19;12:447. doi: 10.1038/s41467-020-20666-3 (PMC7815709; doi:10.1038/s41467-020-20666-3)
Supplement: Supplementary file 6 — Reporting Summary [file 41467_2020_20666_MOESM6_ESM.pdf]

nature research

Corresponding author(s): Maria K. Lehtinen
Last updated by author(s): Nov 28, 2020

Reporting Summary

Nature Research wishes to improve the reproducibility of the work that we publish. This form provides structure for consistency and transparency in reporting. For further information on Nature Research policies, see our Editorial Policies and the Editorial Policy Checklist.

Statistics

For all statistical analyses, confirm that the following items are present in the figure legend, table legend, main text, or Methods section.

n/a	Confirmed	
☐	☒	The exact sample size (n) for each experimental group/condition, given as a discrete number and unit of measurement
☐	☒	A statement on whether measurements were taken from distinct samples or whether the same sample was measured repeatedly
☐	☒	The statistical test(s) used AND whether they are one- or two-sided<br>*Only common tests should be described solely by name; describe more complex techniques in the Methods section.*
☒	☐	A description of all covariates tested
☐	☒	A description of any assumptions or corrections, such as tests of normality and adjustment for multiple comparisons
☐	☒	A full description of the statistical parameters including central tendency (e.g. means) or other basic estimates (e.g. regression coefficient) AND variation (e.g. standard deviation) or associated estimates of uncertainty (e.g. confidence intervals)
☐	☒	For null hypothesis testing, the test statistic (e.g. F, t, r) with confidence intervals, effect sizes, degrees of freedom and P value noted<br>*Give P values as exact values whenever suitable.*
☒	☐	For Bayesian analysis, information on the choice of priors and Markov chain Monte Carlo settings
☒	☐	For hierarchical and complex designs, identification of the appropriate level for tests and full reporting of outcomes
☒	☐	Estimates of effect sizes (e.g. Cohen's d, Pearson's r), indicating how they were calculated

Our web collection on statistics for biologists contains articles on many of the points above.

Software and code

Policy information about availability of computer code

Data collection	Seahorse data collection: Wave software 2.6.0 (Agilent)<br>Confocal imaging: ZEN 2.3 SP1 (Black)<br>MRI: ParaView 6.0.1
Data analysis	Seahorse analysis: Wave software 2.6.0.31 (Agilent);<br>TRAP-seq: STAR 2.4.0 RNA-Seq aligner (with mm10 mouse reference genome); HTSeq software (v 0.6.0); Cufflinks (v 2.2.1); DESeq2 (v 1.26.0); AdvaitaBio iPathway guide v.v1702;<br>Image processing and analysis: ZEN 2.3 (Blue); FIJI (imageJ, 2.0.0); MATLAB R2019b (code included in the manuscript and available on GitHub https://github.com/LehtinenLab/Xu-Fame-2020); IMARIS 9; ParaVew 5.8.0 and ITK-SNAP 3.8.0;<br>Statistics: Prism 8.0

For manuscripts utilizing custom algorithms or software that are central to the research but not yet described in published literature, software must be made available to editors and reviewers. We strongly encourage code deposition in a community repository (e.g. GitHub). See the Nature Research guidelines for submitting code & software for further information.

Data

Policy information about availability of data

All manuscripts must include a data availability statement. This statement should provide the following information, where applicable:

- Accession codes, unique identifiers, or web links for publicly available datasets
- A list of figures that have associated raw data
- A description of any restrictions on data availability

RNA sequencing data are available at: https://www.ncbi.nlm.nih.gov/geo/query/acc.cgi?acc=GSE138970

Field-specific reporting

Please select the one below that is the best fit for your research. If you are not sure, read the appropriate sections before making your selection.

☒ Life sciences ☐ Behavioural & social sciences ☐ Ecological, evolutionary & environmental sciences

For a reference copy of the document with all sections, see nature.com/documents/nr-reporting-summary-flat.pdf

Life sciences study design

All studies must disclose on these points even when the disclosure is negative.

Sample size	sample sizes were determined by power analysis, with mean and deviation estimated from preliminary studies
Data exclusions	In seahorse study (Figure 1h and i), outliers were excluded using ROUT method (Q = 1%). The exclusion was purely based on statistical outcome by ROUT method without any biological consideration. The criteria was pre-established.
Replication	All animal studies were conducted in at least two independent cohorts/litters of mice on different days. All replicates are consistent and presented. We dont' have experiments that did not involve animals.
Randomization	For mice under 7 days old, individuals were randomly assigned to different groups. For mice older with distinguishable male/female features, the mice were randomly distributed into different groups that allowed the two sexes to be equally represented. Littermates were always used as controls, except for adult studies where the mice were purchased as adults. We don't have experiments that did not involve animals.
Blinding	Majority of data was collected by automated software or by core facility staff members. Investigators were not blinded during data collection. For data analysis, in cases where subjective views may influence data outcome (mitochondria size and density quantification, for example), researchers were not able to be blinded due to the obvious difference in experimental groups, but samples from all groups were divided and analyzed by multiple researchers whose measurements reached consistent conclusions independently to rule out subjective bias. Each researcher analyzed equal numbers of samples from each experimental group. For analysis carried out by automated softwares or unlikely to have subjective input (such as Seahorse analysis, mitochondrial distribution, and qPCR), investigators were not blinded.

Reporting for specific materials, systems and methods

We require information from authors about some types of materials, experimental systems and methods used in many studies. Here, indicate whether each material, system or method listed is relevant to your study. If you are not sure if a list item applies to your research, read the appropriate section before selecting a response.

Materials & experimental systems

n/a	Involved in the study	
☐	☒	Antibodies
☒	☐	Eukaryotic cell lines
☒	☐	Palaeontology and archaeology
☐	☒	Animals and other organisms
☒	☐	Human research participants
☒	☐	Clinical data
☒	☐	Dual use research of concern

Methods

n/a	Involved in the study	
☒	☐	ChIP-seq
☒	☐	Flow cytometry
☐	☒	MRI-based neuroimaging

Antibodies

Antibodies used	chicken anti-GFP (Abcam ab13970; 1:1000), mouse anti-Aqp1 (Santa Cruz sc-32737, clone 1/22; 1:100), rabbit anti-NKCC1 (Abcam ab59791; 1:500), rat anti-HA (Roche 11867423001; 1:1000), rabbit anti-pNKCC1 (EMD Millipore ABS1004; 1:1000), rabbit anti-ATP1a1 (Upstate C464.6/05-369; 1:250, goat-anti-klotho (R&D AF1819-sp; 1:200), rabbit anti-GAPDH (Sigma G9545; 1:10000), mouse anti-HA (Abcam ab130275; 1:1000), rabbit anti-CHD4 (Abcam ab72418; 1:2000), rabbit anti-MBD3 (Abcam ab157464; 1:1000), rabbit anti-HDAC1 (Abcam ab7028; 1:2000), mouse anti-HDAC2 (Abcam 51832; 1:2000). Goat anti-mouse HRP secondary antibody (Life

	Tehnologies #31430, 1:10,000). Multiple lots were used for each antibody.
Validation	All primary antibodies were validated with wild-type mouse tissues. For antibodies used in immunohistochemistry, the antibody is validated by: 1) producing signal at correct cellular location (e.g. cytosolic or membrane); 2) not producing signal in the no antibody control. Antibodies validated by this approach include: chicken anti-GFP (Abcam ab13970; 1:1000), mouse anti-Aqp1 (Santa Cruz sc-32737, clone 1/22; 1:100), rabbit anti-NKCC1 (Abcam ab59791; 1:500), rat anti-HA (Roche 11867423001; 1:1000), rabbit anti-pNKCC1 (EMD Millipore ABS1004; 1:1000), abbit anti-CHD4 (Abcam ab72418; 1:2000). For antibodies used in immunoblotting, the antibody is validated by producing bands at the correct molecular weight that match manufacturer description. Antibodies validated by this approach include: rabbit anti-NKCC1 (Abcam ab59791; 1:500), rat anti-HA (Roche 11867423001; 1:1000), rabbit anti-pNKCC1 (EMD Millipore ABS1004; 1:1000), rabbit anti-ATP1a1 (Upstate C464.6/05-369; 1:250, goat-anti-klotho (R&D AF1819-sp; 1:200), rabbit anti-GAPDH (Sigma G9545; 1:10000), mouse anti-HA (Abcam ab130275; 1:1000), rabbit anti-CHD4 (Abcam ab72418; 1:2000), rabbit anti-MBD3 (Abcam ab157464; 1:1000), rabbit anti-HDAC1 (Abcam ab7028; 1:2000), mouse anti-HDAC2 (Abcam 51832; 1:2000).

Animals and other organisms

Policy information about studies involving animals; ARRIVE guidelines recommended for reporting animal research

Laboratory animals	mice: CD-1 (both sexes, embryo E14.5 and E16.5, postnatal day P0, P7, P14, P28, 5-7 weeks, and 2 months); Foxj1:Cre and EGFP-L10a Bacterial Artificial Chromosome (BAC) transgenic lines (both sexes, embryo E16.5 and 2 months adult); loxP-CHD4-loxP transgenic line (both sexes, P7, P14, and P28). Animals were housed in a temperature-controlled room on a 12-hr light/12-hr dark cycle and had free access to food and water.
Wild animals	The study did not involve wild animals
Field-collected samples	The study did not involve field-collected samples. All samples were collected in laboratory.
Ethics oversight	The Boston Children's Hospital IACUC approved all experiments involving mice in this study. Animal Welfare Assurance # A3303-01.

Note that full information on the approval of the study protocol must also be provided in the manuscript.

Magnetic resonance imaging

Experimental design

Design type	MRI was used to image brain ventricles in live mice under anesthesia.
Design specifications	The imaging session was performed on anesthetized mice. No blocks or trials were involved.
Behavioral performance measures	The study did not involve any tasks. The mice were under anesthesia.

Acquisition

Imaging type(s)	Structural
Field strength	7T
Sequence & imaging parameters	Pulse sequences: T2-weighted; imaging type: RARE; TE/TR=60/4000; Ave=8; RARE=4; slice thickness=0.6mm (coronal). 20mm x 20mm field of view. Mtx = 128
Area of acquisition	The whole brain was captured for each scan. Images with visible lateral and third ventricles were included in ventricle volume analysis.

Diffusion MRI ☐ Used ☒ Not used

Preprocessing

Preprocessing software	No preprocessing was involved in the study
Normalization	Ventricle volume was measured as absolute volume (in mm^3).
Normalization template	Data were not normalized
Noise and artifact removal	Fat suppression was used during imaging. No other artifact removal approaches were involved.
Volume censoring	No volume censoring was involved

Statistical modeling & inference

Model type and settings	No model was used. Due to the small size of mouse brain the ventricle volumes were measured by ImageJ.
Effect(s) tested	No task was involved in the study. The MRI was used to measure ventricle volume in anesthetized mice.

Specify type of analysis: ☐ Whole brain ☒ ROI-based ☐ Both

Anatomical location(s)	Lateral ventricles were analyzed. The ventricles appeared white in T2-MRI, and were selected based on the greyscale.

Statistic type for inference (See Eklund et al. 2016)	All statistics were done after the measurements by ImageJ were complete. T-test was used for this study because we only compare ventricle sizes between different groups.
Correction	All samples were used for one comparison only (ventricle volume). No correction was involved.

Models & analysis

n/a	Involved in the study
☒	☐ Functional and/or effective connectivity
☒	☐ Graph analysis
☒	☐ Multivariate modeling or predictive analysis
